# FGF14-AS2 accelerates tumorigenesis in glioma by forming a feedback loop with miR-320a/E2F1 axis

**DOI:** 10.7150/jca.62120

**Published:** 2021-09-03

**Authors:** Peng Zhang, Xueping Gu, Na Zhang, Liang Liu, Xuchen Dong, Haoran Li, Shan Cheng, Suwen Li, Jiaqi Yuan, Yongdong Li, Jun Dong

**Affiliations:** 1 Department of Neurosurgery, The Second Affiliated Hospital of Soochow University, Suzhou 215004, Jiangsu, China.; 2 Rugao Hospital Affiliated to Nantong University, Nantong 226500, Jiangsu, China.; 3 Rugao Clinical College, Jiangsu Health Vocational College, Nantong 226500, Jiangsu, China.; 4 Medical College of Soochow University, Suzhou 215123, Jiangsu, China.

**Keywords:** glioma, lncRNA, FGF14-AS2, miR-320a, E2F1

## Abstract

Glioma is the most common primary tumour in the central nervous system in adults, and at present, there is no effective treatment to cure this malignancy. Long noncoding RNAs (lncRNAs) are closely related to tumour progression and have attracted increasing attention in tumour research. However, the role of lncRNA FGF14-AS2 in glioma tumorigenesis has not been determined. In the present study, we found that FGF14-AS2 expression was significantly elevated in glioma tissues and was associated with poor survival in glioma patients. Silencing FGF14-AS2 inhibited the proliferation, migration and invasion ability of glioma cells. *In vivo* assay showed that silencing FGF14-AS2 led to inhibition of tumour growth. In addition, FGF14-AS2 was observed to promote glioma progression via the miR-320a/E2F1 axis. Moreover, E2F1 could bind to the promoter region of FGF14-AS2, thereby enhancing FGF14-AS2 expression. In conclusion, FGF14-AS2 could accelerate tumorigenesis of glioma by forming a feedback loop with the miR-320a/E2F1 axis which suggested that FGF14-AS2 could serve as a therapeutic target for glioma.

## Introduction

Glioma, which accounts for approximately 40-50% of all central nervous system (CNS) tumours, is the most common primary CNS tumour in adults 1, 2. The clinical symptoms of glioma primarily include headache, vomiting, epilepsy, loss of neurological function, disturbance of consciousness and cerebral hernia caused by the tumour mass effect, which seriously threaten the life of glioma patients 3. At present, glioma is difficult to cure by surgery and existing adjuvant treatments. Therefore, it is of considerable practical significance and theoretical value to explore the molecular mechanisms governing gliomagenesis.

Long noncoding RNAs (lncRNAs) represent a class of noncoding RNA molecules that are longer than 200 bp 4, 5. Because these RNAs do not have the potential to encode proteins, they have been considered to be the “dark matter” of the genome 6. In 2007, Rinn, et al. elucidated the structure and function of a well-known lncRNA, HOTAIR 7. Subsequently, lncRNAs have continued to attract the attention of many scholars, and lncRNAs have become a heavily studied topic in human disease research. The current evidence shows that lncRNAs play a multilevel regulatory role in various tumours. In nucleus, lncRNAs can combine with transcription factors (TFs) to regulate the transcription of related genes and subsequently trigger a series of biological effects 8. In cytoplasm, lncRNAs can bind to specific signal proteins located in the cytoplasm and affect their expression, thereby regulating tumour progression 9. On the other hand, lncRNAs can absorb another type of noncoding RNA, microRNA (miRNA), in the cytoplasm and affect the miRNA-mediated regulation of downstream messenger RNA (mRNA) molecules, thereby regulating tumour progression 10, 11. LncRNA fibroblast growth factor 14 antisense RNA 2 (FGF14-AS2), located on chromosome 13, is transcribed from the fibroblast growth factor 14 (FGF14) gene. FGF14-AS2 was reportedly related to the prognosis of breast cancer 12 and inhibited breast cancer progression by sponging miR-370-3p or miR-205 13, 14. In subsequent years, there has been no research on the relationship between FGF14-AS2 and human disease, except that FGF14-AS2 sponges miR-1288-3p to inhibit the proliferation of colorectal cancer 15.

MiRNAs, another important type of noncoding RNA, can regulate gene expression at the posttranscriptional level by binding to the 3′-untranslated region (3′-UTR) of mRNA molecules and are thus involved in the regulation of various cellular processes 16. Accumulating evidence has indicated that miRNAs can be absorbed by lncRNAs, leading to a weakened regulatory effect on target mRNAs 17. MiR-320a is involved in the development of some malignant tumours, including mesothelioma, cholangiocarcinoma and glioma 18-20. Although some studies have indicated that miR-320a may function as a tumour suppressor in glioma, its role and regulatory network in glioma have not been fully elucidated to date.

The E2Fs, a family of transcription factors, are closely related to the balance of the cell cycle. E2F1, as an important E2F member, has been reported to be closely related to breast cancer, hepatocellular carcinoma and lung cancer 21-23. Studies have also suggested that E2F1 may function as an oncogene in glioma 24-26; however, the exact role and mechanism of E2F1 in the lncRNA-miRNA network has not been determined.

In current study, we observed that FGF14-AS2 was upregulated in glioma tissues and cell lines. The results of assays suggested that FGF14-AS2 promotes gliomagenesis. Moreover, bioinformatic analysis, RNA pull-down assays, RIP assays, luciferase reporter assays and cell function assays confirmed that FGF14-AS2 could function as a sponge for miR-320a and that miR-320a could target E2F1. In addition, E2F1 was found to bind to the FGF14-AS2 promoter region and enhance FGF14-AS2 expression. Taken together, the results of this study demonstrated that E2F1/FGF14-AS2/miR-320a formed a feedback loop to regulate the progression of glioma and suggested that FGF14-AS2 could serve as a biomarker for glioma.

## Methods

### Ethics statement

This study was approved by the Ethics Committee of the Second Affiliated Hospital of Soochow University. Animal experiments were conducted in accordance with the Guide for the Care and Use of Laboratory Animals of Second Affiliated Hospital of Soochow University and complied with the ARRIVE guidelines.

### Clinical specimens

Thirty glioma tissues and paired adjacent normal brain tissues were collected from glioma patients who were admitted to the Department of Neurosurgery, Second Affiliated Hospital of Soochow University, from June 2012 to July 2017. All tissues were stored in liquid nitrogen immediately after surgical resection. Written informed consent was obtained from all the patients.

### Cell culture and transfection

T98G and LN229 cells were purchased from American Type Culture Collection (VA, USA). Normal human astrocytes (NHAs) were purchased from JENNIO Biological Technology (Guangzhou, China). Cells were cultured in Dulbecco's modified Eagle's medium (DMEM, Gibco, NY, USA) supplemented with 10% foetal bovine serum (FBS, ScienCell, LA, USA) and maintained in an incubator containing 5% CO_2_ at 37 °C. Synthetic short interfering RNA (shRNA), miR-320a mimic and miR-320a inhibitor were purchased from GenePharma (Shanghai, China). Lipofectamine 3000 (Invitrogen, CA, USA) was utilized to perform the transfection assays.

### Reverse transcription quantitative polymerase chain reaction (qRT-PCR)

The primers employed in this assay were purchased from GenePharma. The qRT-PCR procedures have been described in detail in a previous study^27^. In brief, RNA was extracted from tissues and cells with TRIzol (Invitrogen). After the concentration was determined, ABI Prism 7700 (Applied Biosystems, MA, USA) was used to perform qRT-PCR. GAPDH and U6 were used as reference genes. Finally, the relative expression levels of FGF14-AS2, miR-320a and E2F1 were measured by the 2^-ΔΔCt^ method. The primers used were shown below: FGF14-AS2: forward, 5'-ATTAC CGAGG GGTTC CACGC-3' and reverse, 5'-GGTGT CCGGG ACCAG AAAGT-3'; miR-320a: forward, 5'-AAAAG CTGGG TTGAG AGGGC GA-3' and reverse, 5'- GCGAG CACAG AATTA ATACG AC-3'; E2F1: forward, 5'- CCCAT CCCAG GAGGT CACTT-3' and reverse, 5'- CTGCA GGCTC ACTGC TCTC-3'.

### Western blotting

The Western blot assay procedures were described in our previous study 27. Total protein was extracted with RIPA buffer (KenGEN, Shanghai, China). After separation by sodium dodecyl sulphate polyacrylamide gel electrophoresis, the proteins were transferred to polyvinylidene fluoride membranes followed by sealing with skim milk. Next, the membrane was incubated with primary antibody and secondary antibody. After washing with TBST, the membrane was immersed in an Image Quant LAS 4000 mini (GE, USA). The antibodies against to GAPDH (60004-1-Ig) and E2F1 (66515-1-Ig) were purchased from Proteintech (IL, USA).

### Luciferase reporter assay

T98G and LN229 cells were cultured in 6-well plates. The fragments of the E2F1 3′-UTR and FGF14-AS2 containing binding sites for miR-320a were inserted into the pMIR-REPORT vector. The assay was performed according to the manufacturer's protocol, which has been described in our previous study 28. The relative luciferase activity was normalized to Renilla luciferase activity.

### RNA pull-down assay

RNA pull-down assay was carried out with a Pierce Magnetic RNA-Protein Pull-Down Kit (Thermo Scientific, USA) according to the manufacturer's protocol. The biotinylated FGF14-AS2 or miR-320a probes were mixed with cellular protein extracts and subsequently cultured with magnetic beads. Next, qRT-PCR was performed to detect the expression of the target miRNAs and FGF14-AS2 in the pull-down assay 29.

### RNA Immunoprecipitation (RIP) assay

RIP assay was conducted with the Magna RIP RNA-Binding Protein Immunoprecipitation Kit (Merck Millipore, USA). After cells were harvested from RIP lysis buffer, anti-Ago2 and anti-IgG were used for immunoprecipitation. Next, the precipitates were recovered by beads, and qRT-PCR was performed to determine the relative expression level of FGF14-AS2.

### 5-Ethynyl-20-deoxyuridine (EdU) assay

Cell proliferative ability was evaluated by EdU assay, which was performed with an EdU cell proliferation assay kit (RiboBio, Guangzhou, China) according to the manufacturer's protocol. Cells were incubated with EdU solution for 2 h in an incubator containing 5% CO_2_ at 37 °C and later fixed in paraformaldehyde (4%) for 15 min, then, cells were treated with Triton X-100 (0.4%, KeyGen, Shanghai, China) for 10 min and incubated with Apollo reagent for 30 min. Subsequently, the nuclei were stained with 4′,6-diamidino-2-phenylindole (DAPI, Beyotime, Shanghai, China) for 5 min. Finally, a fluorescence microscope was employed to capture the images.

### Transwell assay

Transwell assay was performed as described in our previous study 30. For the Transwell invasion assay, the chambers (Millipore, Germany) were precoated with 50 µl Matrigel (Matrigel: DMEM = 1:9, BD, USA). Next, cells (100,000) were suspended in 1 ml FBS-free DMEM, and 200 μl cell suspension was added to the upper chambers. Next, DMEM (600 μl) containing 10% FBS was added to the lower chamber as a chemotactic agent. After incubation for 24 h, cells that remained in the upper chamber were removed, and the invaded cells were fixed with paraformaldehyde (4%) and stained with crystal violet. Finally, the invaded cells were counted under a microscope (Olympus, Tokyo, Japan). For the Transwell migration assay, the procedure was the same as that employed for the Transwell invasion assay, except that the chamber was not precoated with Matrigel.

### Tumour xenograft in nude mice

Eight (four each group) Balb/c female nude mice (4-6 weeks old) were purchased from Beijing Laboratory Animal Center (Beijing, China). LN229 cells (1×10^8^) in the logarithmic phase were injected subcutaneously into the shoulder of the forelimb of the nude mice. Twenty-one days later, the mice were sacrificed. The tumours that had formed were removed, weighed and measured.

### Statistical analysis

The data were analysed by GraphPad 8.0 and expressed as the mean ± standard deviation. The difference between two groups was measured by Student′s t-test. P-values < 0.05 were considered to indicate significance. All assays were performed three times independently.

## Results

### FGF14-AS2 is elevated in glioma tissues and is associated with poor survival in glioma patients

**Figure 1:**
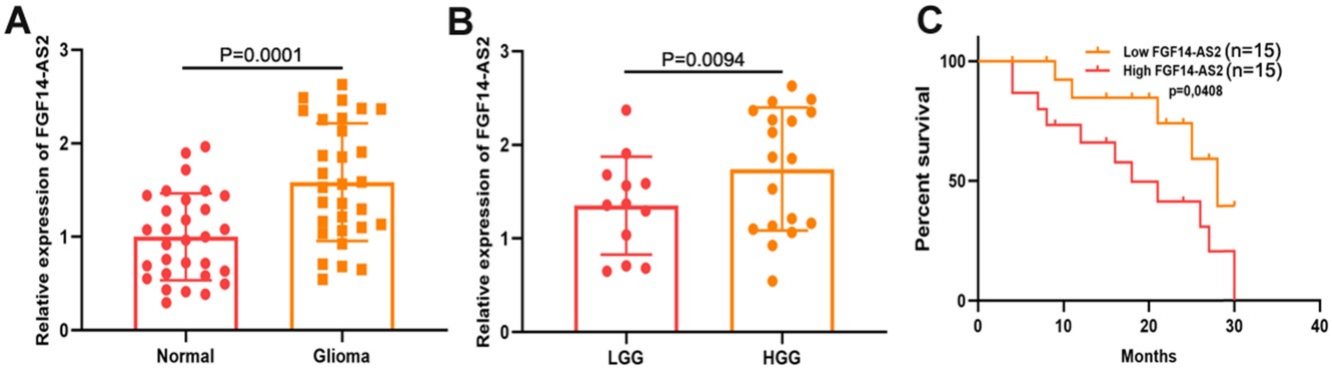
** FGF14-AS2 is elevated in glioma tissues and associated with the survival of glioma patients. (A)** Relative expression of FGF14-AS2 in glioma tissues and paired adjacent normal brain tissues detected by qRT-PCR. **(B)** Relative expression of FGF14-AS2 in low-grade glioma and high-grade glioma detected by qRT-PCR. **(C)** Kaplan-Meier analysis of glioma patients with high or low expression of FGF14-AS2. * P<0.05, ** P<0.01.

To measure the expression levels of FGF14-AS2 in glioma and paired adjacent normal brain tissues, qRT-PCR was performed. The results indicated that the FGF14-AS2 expression level was higher in glioma tissues than in paired adjacent normal brain tissues **(Fig. 1A)**. According to the World Health Organization's glioma classification standards, these clinical specimens were divided into low-grade glioma (LGG) and high-grade glioma (HGG). The qRT-PCR results showed that FGF14-AS2 was overexpressed in HGG compared with LGG **(Fig. 1B)**. In addition, Kaplan-Meier analysis showed that high FGF14-AS2 expression indicated poor overall survival **(Fig. 1C)**. These data suggested that FGF14-AS2 may be involved in the progression of glioma.

### FGF14-AS2 promotes the proliferation, migration and invasion of glioma cells *in vitro* and accelerates glioma growth* in vivo*

**Figure 2:**
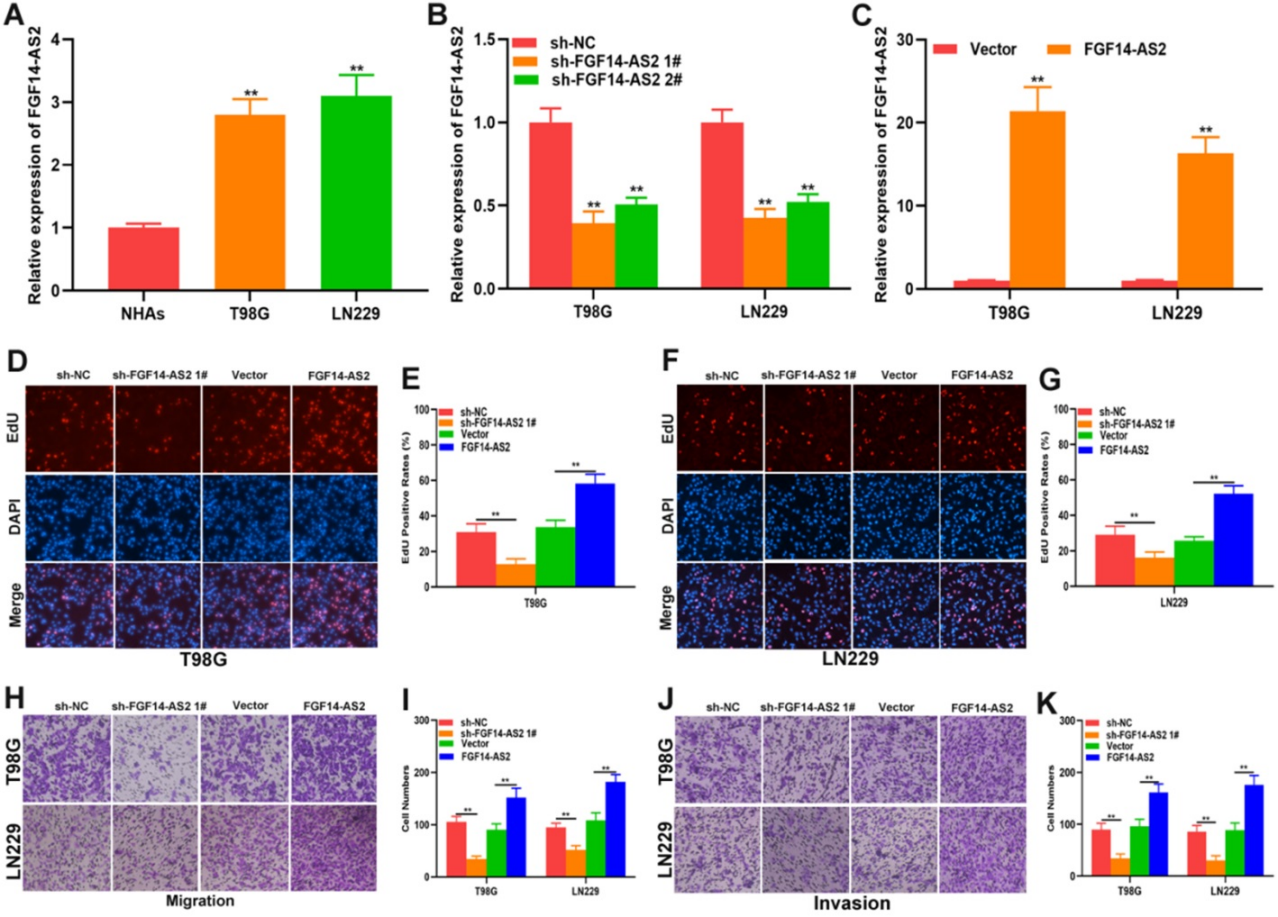
** FGF14-AS2 promotes the proliferation, migration and invasion of glioma cells *in vitro*. (A)** Expression of FGF14-AS2 in NHAs, T98G and LN229 detected by qRT-PCR. **(B-C)** FGF14-AS2-knockdown and FGF14-AS2-overexpressing T98G and LN229 cell lines were established (sh-NC: knockdown control cells; sh-FGF14-AS2: FGF14-AS2 knockdown cells; vector: overexpression control cells; FGF14-AS2: FGF14-AS2-overexpressing cells). **(D-G)** EdU assay of the proliferation abilities of FGF14-AS2-knockdown and FGF14-AS2-overexpressing T98G and LN229 cells. **(H-K)** Transwell assay of the migration and invasion capabilities of FGF14-AS2-knockdown and FGF14-AS2-overexpressing T98G and LN229 cells. * P<0.05, ** P<0.01.

**Figure 3:**
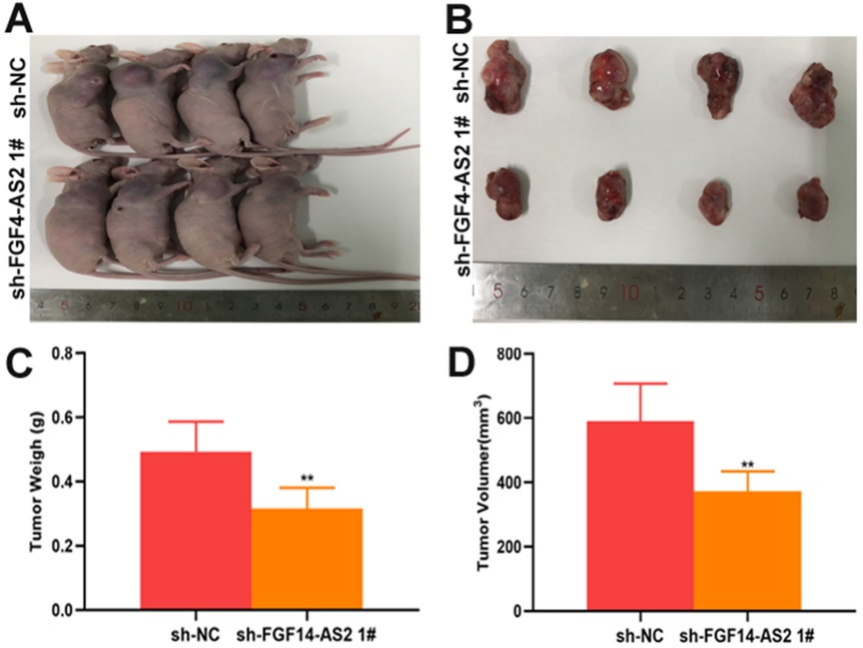
** Silencing FGF14-AS2 inhibit tumour growth *in vivo*. (A-B)** The tumours formed in the nude mice. **(C-D)*** In vivo* assay illustrated that the tumour weight and volume were decreased after FGF14-AS2 knockdown. * P<0.05, ** P<0.01.

To investigate the function of FGF14-AS2 in glioma, normal human astrocytes and glioma cell lines (T98G and LN229) were cultured. The expression of FGF14-AS2 in the three cell lines was detected by qRT-PCR, and the results showed that compared with NHAs, FGF14-AS2 was upregulated in T98G and LN229 cells **(Fig. 2A)**. Next, loss- and gain-of-function assays were conducted by using T98G/LN229 cells transfected with sh-FGF14-AS2 or FGF14-AS2 overexpression plasmids. FGF14-AS2 was silenced or strengthened after sh-FGF14-AS2 or FGF14-AS2 overexpression plasmid transfection, respectively **(Fig. 2B, C)**. The EdU assay illustrated that FGF14-AS2 knockdown was reduced, whereas FGF14-AS2 overexpression promoted the proliferation of T98G and LN229 cells **(Fig. 2D-G).** The results of Transwell assays demonstrated that silencing FGF14-AS2 suppressed the migration and invasion capacity of T98G and LN229 cells; however, FGF14-AS2 overexpression had the opposite effect **(Fig. 2H, K)**. Moreover, an *in vivo* assay showed that knockdown of FGF14-AS2 decreased the weight and volume of the tumours formed in nude mice **(Fig. 3A-D)**. These findings indicated that FGF14-AS2 promoted the malignant progression of glioma *in vitro* and *in vivo*.

### FGF14-AS2 functions as a sponge of miR-320a in glioma

**Figure 4:**
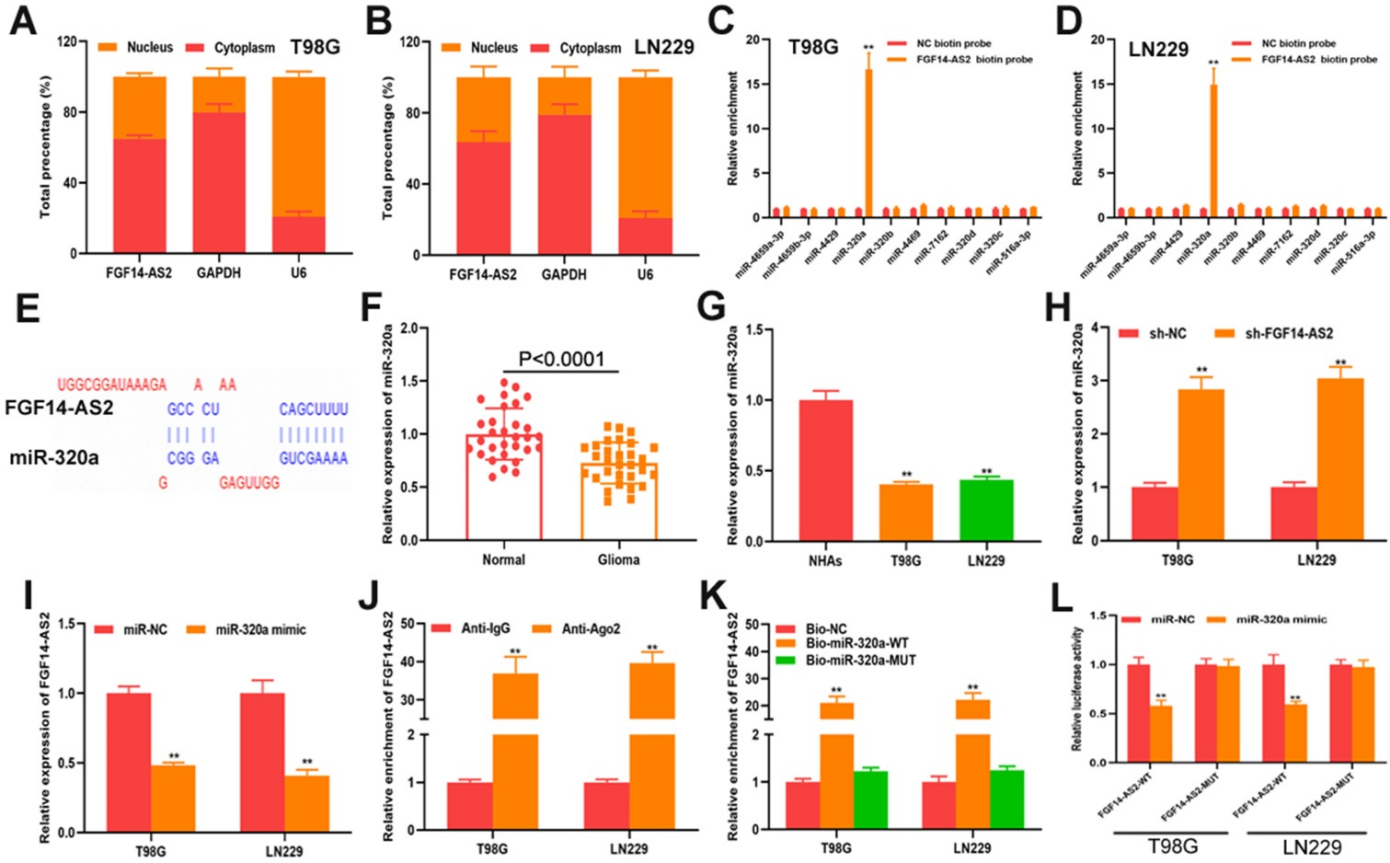
** FGF14-AS2 functions as s sponge of miR-320a in glioma. (A-B)** Subcellular fractionation assay showed that FGF14-AS2 was mainly located in the cytoplasm. **(C-D)** RNA pull-down assay was performed to explore the potential interaction between candidate miRNAs and FGF14-AS2. **(E)** The putative binding sites between FGF14-AS2 and miR-320a. **(F)** Relative expression of miR-320a in glioma tissues and paired adjacent normal brain tissues detected by qRT-PCR. **(G)** Relative expression of miR-320a in NHAs, T98G and LN229 detected by qRT-PCR. **(H)** Relative expression of miR-320a in T98G and LN229 cells after FGF14-AS2 knockdown. **(I)** Relative expression of FGF14-AS2 in T98G and LN229 cells after miR-320a overexpression. **(J)** RIP assays revealed that FGF14-AS2 had the potential to bind to Ago2. **(K)** RNA pull-down assay validated the interaction between FGF14-AS2 and miR-320a. **(L)** Dual-luciferase reporters showed that miR-320a mimics decreased the luciferase activity of the vector containing FGF14-AS2-WT. * P<0.05, ** P<0.01.

To elucidate the underlying mechanism of FGF14-AS2 in glioma progression, a subcellular fractionation assay was performed to detect the distribution of FGF14-AS2 in T98G and LN229 cells. The results showed that FGF14-AS2 was higher in the cytoplasm than in the nucleus, which suggested that FGF14-AS2 could act as a sponge of miRNAs **(Fig. 4A, B)**. Data from the online database lncBase (www.microrna.gr/LncBase) showed that 40 miRNAs could be absorbed by FGF14-AS2. From these candidates, 10 miRNAs with the highest score were screened out for further analysis. RNA pull-down assay indicated that miR-320a was the only miRNA that could be clearly enriched by biotin-labelled FGF14-AS2 **(Fig. 4C, D)**. The putative binding site between FGF14-AS2 and miR-320a was shown in **Figure 4E**. Next, qRT-PCR was performed to detect miR-320a expression in glioma tissues and cell lines. The data showed that miR-320a was downregulated in glioma tissues and cell lines compared with normal brain tissues and normal human astrocytes, respectively **(Fig. 4F, G)**. In T98G and LN229 cells, silencing FGF14-AS2 resulted in upregulation of miR-320a, whereas overexpressing miR-320a decreased FGF14-AS2 expression **(Fig. 4H, I)**. In addition, RIP assay and RNA pull-down assay revealed that FGF14-AS2 could bind to Ago2, and that FGF14-AS2 could be significantly enriched by biotin-labelled miR-320a **(Fig. 4J, K)**. Finally, a luciferase reporter assay was performed, and the results showed that miR-320a could reduce the luciferase activity of FGF14-AS2-WT but not FGF14-AS2-MUT **(Fig. 4L)**. Taken together, these results demonstrated that FGF14-AS2 could function as a sponge of miR-320a.

### E2F1 is the direct target of miR-320a

**Figure 5:**
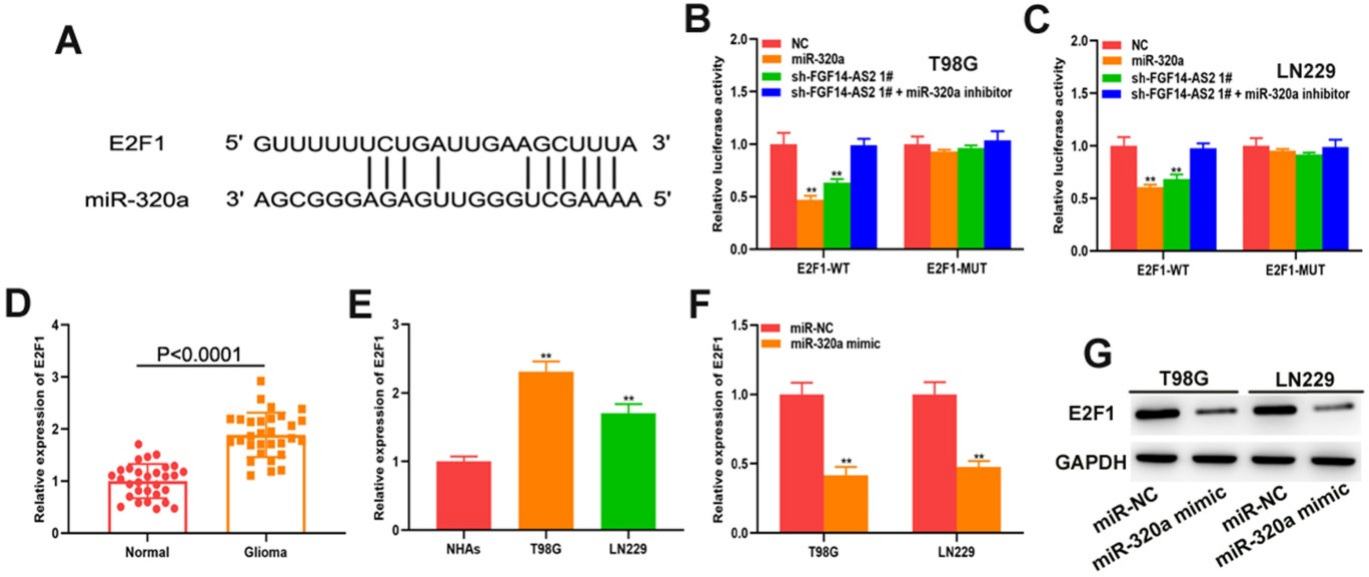
** E2F1 is the direct target of miR-320a. (A)** The putative binding sites between E2F1 and miR-320a. **(B,C)** Luciferase reporter assay of cells transfected with E2F1-WT or E2F1-MUT reporter together with miR-320a, FGF14-AS2 shRNA or FGF14-AS2 shRNA plus miR-320a inhibitor. **(D)** Relative expression of E2F1 in glioma tissues and paired adjacent normal brain tissues detected by qRT-PCR. **(E)** Relative expression of E2F1 in NHAs, T98G and LN229 detected by qRT-PCR. **(F,G)** Relative expression of E2F1 in T98G and LN229 cells after miR-320a overexpression detected by qRT-PCR and Western blotting. * P<0.05, ** P<0.01.

To explore the underlying mechanism governing the regulatory pathways of these RNAs further, the online database starBase was used to predict the potential mRNAs that could be directly targeted by miR-320a, from which E2F transcription factor 1 (E2F1) attracted our attention. The putative binding site between miR-320a and E2F1 was shown in **Figure 5A**. Luciferase reporter assay showed that both the miR-320a mimic and sh-FGF14-AS2 reduced the luciferase activity of E2F1-WT but not E2F1-MUT** (Fig. 5B, C).** The expression of E2F1 in glioma tissues and glioma cell lines was measured, and the results showed that E2F1 was upregulated in glioma tissues and cell lines compared with normal brain tissues and normal human astrocytes, respectively **(Fig. 5D, E)**. Additionally, qRT-PCR and Western blot analysis showed that miR-320a could decrease the expression of E2F1 **(Fig. 5F, G)**. Taken together, these results demonstrated that E2F1 is the direct target of miR-320a.

### FGF14-AS2 promotes the proliferation, migration and invasion of glioma cells via the miR-320a/E2F1 axis

**Figure 6:**
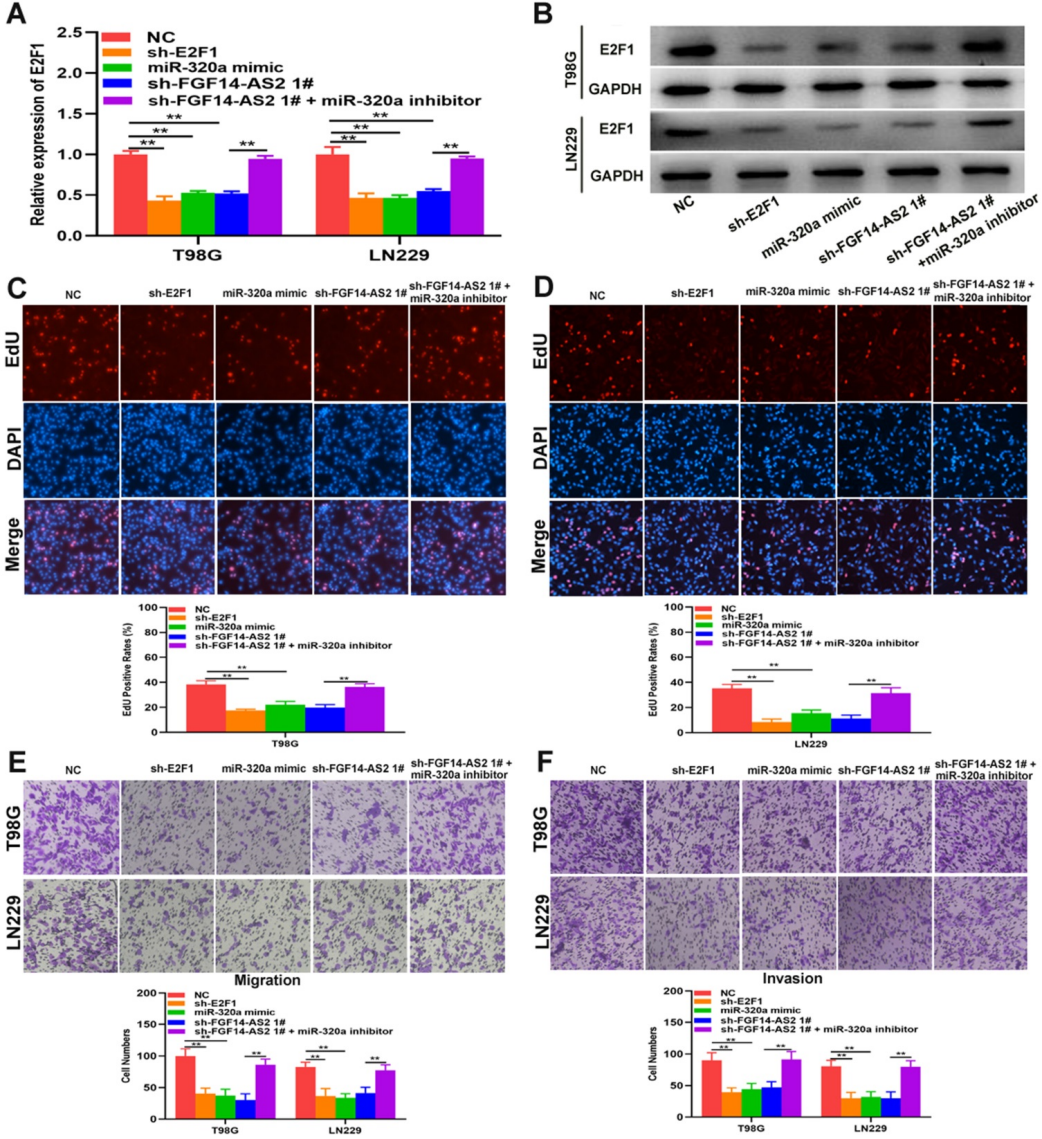
** FGF14-AS2 promotes the proliferation, migration and invasion of glioma cells via the miR-320a/E2F1 axis. (A,B)** E2F1 expression changes in E2F1 shRNA, miR-320a mimic, FGF14-AS2 shRNA, FGF14-AS2 shRNA plus miR-320a inhibitor-transfected T98G and LN229 cells detected by qRT-PCR and Western blotting. **(C,D)** The proliferation capacity of E2F1 shRNA, miR-320a mimic, FGF14-AS2 shRNA, FGF14-AS2 shRNA plus miR-320a inhibitor transfected T98G and LN229 cells was assessed by EdU assay. **(E,F).** The migration and invasion capacity of E2F1 shRNA, miR-320a mimic, FGF14-AS2 shRNA, FGF14-AS2 shRNA plus miR-320a inhibitor-transfected T98G and LN229 cells was assessed by Transwell assay. * P<0.05, ** P<0.01.

To perform a more rigorous analysis, E2F1 and FGF14-AS2 were silenced, whereas miR-320a was overexpressed, in T98G and LN229 cells by shRNAs and miR-320a mimic, respectively **(Fig. 6A, B)**. EdU assay showed that sh-E2F1, miR-320a mimic and sh-FGF14-AS2 could reduce the proliferative ability of T98G and LN229 cells, whereas the inhibitory effect caused by sh-FGF14-AS2 could be partly alleviated by the miR-320a inhibitor **(Fig. 6C, D)**. Similarly, Transwell assay showed that the migration and invasion ability was impaired by sh-E2F1, miR-320a mimic and sh-FGF14-AS2, and the effect of sh-FGF14-AS2 on migration and invasion could be reversed by the miR-320a inhibitor **(Fig. 6E, F)**. Taken together, these results demonstrated that FGF14-AS2 promotes the proliferation, migration and invasion of glioma cells via the miR-320a/E2F1 axis.

### E2F1 promotes the transcriptional expression of FGF14-AS2

**Figure 7:**
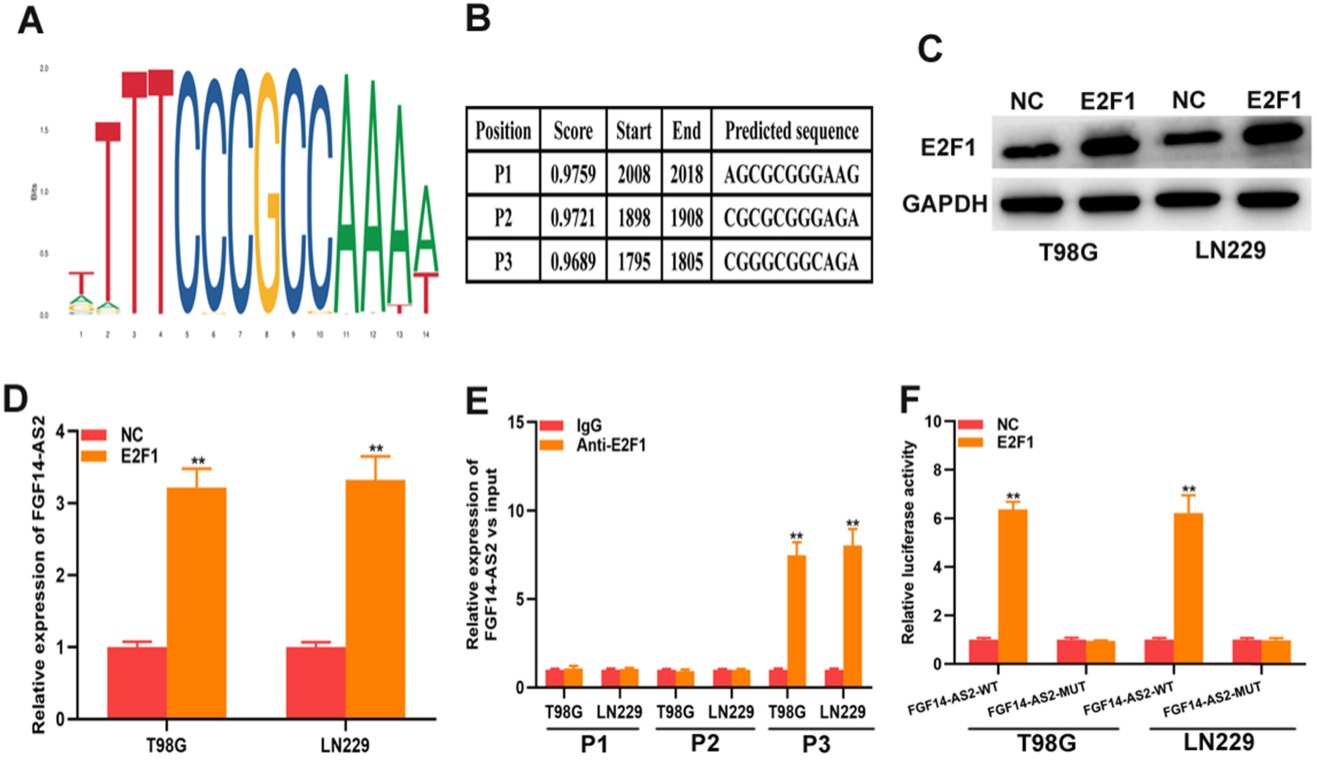
** E2F1 promotes the transcriptional expression of FGF14-AS2. (A,B)** Putative E2F1 binding site in the FGF14-AS2 promoter region predicted by JASPAR. **(C)** E2F1 protein expression level in T98G and LN229 cells transfected with E2F1 recombinant plasmid. **(D)** Relative expression of FGF14-AS2 in T98G and LN229 cells transfected with the E2F1 recombinant plasmid. **(E)** ChIP analysis showed the binding affinity in the predicted binding sites. **(F)** A luciferase reporter assay revealed the activity of E2F1 and FGF14-AS2-WT or FGF14-AS2-MUT. * P<0.05, ** P<0.01.

As a well-known transcription factor, E2F1 could regulate the transcription of many genes. In current study, whether E2F1 could regulate the transcription of FGF14-AS2 need to be further investigated. The data from Jaspar (http://jaspar.genereg.net) indicated that there are sites in the promoter region of FGF14-AS2 to which E2F1 could bind **(Fig. 7A, B)**. The E2F1 overexpression plasmid was transfected into T98G and LN229 cells, and the effectiveness of E2F1 expression was testified by Western blot **(Fig. 7C)**. qRT-PCR demonstrated that overexpression of E2F1 was capable of elevating FGF14-AS2 expression level **(Fig. 7D)**. Besides, the ChIP assay showed that the affinity of the FGF14-AS2 promoter for E2F1 was considerably higher than that for IgG (P3)** (Fig. 7E).** Finally, luciferase reporter assay indicated that wild‐type FGF14-AS2 showed high activity with E2F1 **(Fig. 7F)**. In conclusion, the above data indicated that E2F1 upregulated FGF14-AS2 expression by binding to its promoter region.

## Discussion

Long noncoding RNAs are important components of the transcriptome. Since Salmena proposed that lncRNAs could function as molecular sponges to adsorb miRNAs and thus regulate the expression of mRNAs, the “lncRNA-miRNA-mRNA” axis has been determined to play a regulatory role in many human diseases, including glioma^31^. With the progress of transcriptome sequencing technology, an increasing number of lncRNAs have been discovered, and an increasing number of lncRNAs have been proven to be closely related to a variety of biological processes, such as transcriptional regulation of genes, epigenetic regulation, chromatin modification and ontogenetic regulation 32, 33. To date, an increasing number of studies have documented that abnormal expression of lncRNAs is closely associated with various tumours, some of which are considered to be important potential therapeutic targets for tumour therapy 34. Numerous lncRNAs have also been reported to be involved in the malignant progression of glioma. For example, GAS5 and HOTAIR were dysregulated in gliomas and related to the proliferation, migration, invasion and chemosensitivity of glioma cells 28, 35-37. FGF14-AS2 was reported to function as a suppressor gene in breast cancer and colorectal cancer 13, 15; however, its role in glioma has not been reported to date. The current study showed that FGF14-AS2 was upregulated in glioma tissues and cells, more interestingly, high expression of FGF14-AS2 predicted a poor prognosis in glioma. Additionally, functional assays showed that FGF14-AS2 could strengthen the proliferation, migration and invasion ability of glioma cells.

Subcellular localization showed that FGF14-AS2 was more highly expressed in the cytoplasm, which means that FGF14-AS2 could function as a sponge for miRNAs. Bioinformatic analyses showed that there are 40 miRNAs that have sites that could be bound by FGF14-AS2. Based on RIP assay, miR-320a was screened out for further study. Dysregulated miR-320a has been determined to play a critical role in various cancers, such as cervical cancer 38, melanoma 39, nasopharyngeal carcinoma 40 and retinoblastoma 41. In keeping with the findings of prior studies, our results showed that compared with normal brain tissues and normal human astrocytes, miR-320a was downregulated in glioma tissues and cell lines, respectively. Combined with the results of RNA pull-down assay and luciferase reporter assay, miR-320a was considered to be the sponged target of FGF14-AS2.

In addition, the data from an online database indicated that miR-320a could bind to the 3′-UTR of E2F1, which is a well-known transcriptional activator that has been determined to be extensive involved in tumour progression in such cancers as bladder cancer 42 breast cancer 43, and lung cancer 44. In our study, we found that E2F1 was upregulated in glioma tissues and cells and could be directly suppressed by miR-320a. Functional assays further disclosed that E2F1 acts as an oncogene in glioma. A luciferase reporter assay showed that both FGF14-AS2 sh-RNA and miR-320a mimic decreased the luciferase activity of E2F1-WT, and the inhibitory effect of FGF14-AS2 sh-RNA was restored by the miR-320a inhibitor. Moreover, qRT-PCR and Western blot analysis showed that knockdown of FGF14-AS2 decreased E2F1 expression, and this effect could be partly abrogated by the miR-320a inhibitor. Moreover, functional experiments indicated that the effect of sh-FGF14-AS2 on the proliferation, migration and invasion of glioma could be abolished by miR-320a inhibitor. Taken together, these results suggested that FGF14-AS2 acts as a ceRNA to regulate the miR-320a/E2F1 axis and is thus involved in the malignant progression of glioma.

As a transcription factor, we wondered whether E2F1 could promote FGF14-AS2 transcription. Through analysis with the online tools JASPAR and UCSC, sites on the promoter region of FGF14-AS2 that could be bound by E2F1 were identified. Furthermore, the effective binding sites between FGF14-AS2 and E2F1 were supported by qRT-PCR, ChIP and luciferase reporter assays.

In conclusion, this study demonstrated that FGF14-AS2 promotes tumour progression in glioma *in vitro* and *in vivo*. Mechanistically, E2F1 binds to the promoter region of FGF14-AS2, forming the FGF14-AS2/miR-320a/E2F1 feedback loop which may offer potential target for precision therapy against glioma.
